# Implantable Loop Recorder Identifies Sustained Ventricular Tachycardia in a Pediatric Patient Despite Negative EP Study: A Case Report

**DOI:** 10.1155/crpe/9080260

**Published:** 2025-09-30

**Authors:** Jia Yue Liu, Mohammed T. Numan

**Affiliations:** Department of Pediatric Cardiology, The University of Texas Health Science Center at Houston, Houston, Texas, USA

## Abstract

Experience of loop recorders in pediatric patients is limited. We report using a loop recorder to detect ventricular arrhythmias in a 16-year-old female who had years of palpitations despite inconclusive noninvasive and invasive electrophysiological testing.

## 1. Background

Patients with family history of sudden death can be a clinical challenge for cardiologists. Some of those patients will present with nonspecific symptom of palpitations. Subsequently, preliminary noninvasive studies can be inconclusive. This can lead to the performance of an invasive electrophysiological diagnostic study which also can turn to be inconclusive. The unresolved complaint of palpitation will raise the question “what else to do” in this higher risk group. We describe a typical such-case scenario that had palpitations for 6 years with multiple investigations by several cardiologists.

## 2. Clinical Vignette

A 16-year-old female with history of attention-deficit hyperactivity disorder, anxiety, and migraine headaches presented with complaints of palpitations to her local cardiologist six years prior to her referral to our electrophysiology (EP) service. At her initial presentation, her 12-lead ECG showed normal sinus rhythm and intervals. A Holter monitor was obtained and revealed no arrhythmia. Later, she began experiencing palpitations daily in addition to excessive sweating associated with these episodes. These episodes last for minutes and could occur with or without vigorous activity. The patient had normal cardiac structures and function with no evidence of cardiomyopathy by echocardiography.

Another Holter was placed by the local cardiologist and revealed infrequent isolated polymorphic ventricular beats with long couplet. At our center, exercise stress testing performed with no induced arrhythmia. With a detailed family history, mother reported that her maternal grandmother's cousin passed away suddenly in their early 30s, of unknown cause of death. Because of this significant family history and the ventricular ectopy on the Holter monitor, EP study was performed, and in the same time, genetic testing for arrhythmia mutations was obtained. Cardiac MRI was performed which did not show arrhythmogenic right ventricular dysplasia. There was no induced ventricular ectopy during EP study with aggressive ventricular protocol and isoprenaline infusion. The genetic test revealed two heterozygous variants of uncertain significance in *AKAP9* and *SCNA8A*. After discussion with the patient's family, we placed an implantable loop recorder (ILR). Family did not wish to have a repeat EP study.

**Figure 1:**
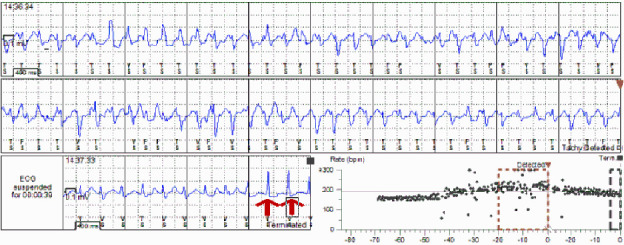
ILR strip showing sustained polymorphic ventricular tachycardia with spontaneous conversion to sinus rhythm (red arrows).

**Figure 2:**
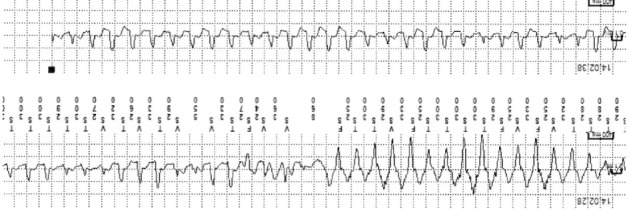
ILR strip showing sustained polymorphic ventricular tachycardia.

After 1 month of ILR implant, the monitor demonstrated two episodes of sustained polymorphic ventricular tachycardia (PVT) (Figures 1 and 2). After discussion of the potential risk of sudden death, the patient and her family decided to proceed with placement of an implantable cardioverter defibrillator (ICD).

Over 1-year follow-up, there was no delivered therapy by the ICD upon interrogations.

## 3. Discussion

This case demonstrates the diagnostic limitations of ambulatory monitors and EP studies in risk-stratification for ventricular arrhythmia in moderate–high-risk population. With negative exercise stress test, ambulatory monitor, and invasive EP study, probably most of the physicians will be satisfied not to pursue further intervention since collectively these studies do not reveal significant arrhythmia. The significant diagnostic power of ILR in such clinical scenario should be entertained by electrophysiologists.

The use of ILRs in adults with syncope is well established with sparse cases in pediatrics [1–4]. The role ILRs in pediatric practice is still under-utilized. Overall, the yield of 24-h Holter ECG monitoring is low sensitivity and specificity in detecting relevant arrhythmias [5, 6]. While event monitors show better correlation with patients' symptoms than Holter monitors, the sensitivity is still low [7]. One study showed that event monitors are useful for diagnosis for 48% of children and adolescents with suspected arrhythmia with the remainder of patients failed to transmit a legible ECG during symptoms [7]. This is comparable to two similar pediatric studies [8, 9]. Even invasive percutaneous cardiac EP procedure also has a low sensitivity of 50% with a high specificity of 75%–95% [10].

Currently, there are no guidelines for the use of ILRs in pediatric patients. The American College of Cardiology guidelines for ambulatory ECG suggest that the ILR can be particularly useful in monitoring patients who have infrequent symptoms [11]. The European Society of Cardiology guidelines for managing syncope advise that the conventional investigation and that the ILR are a last resort—except in patients with structural heart disease, exercise-induced syncope, a family history of SCD, chest pain or palpitations, or ECG results that suggest a risk of arrhythmic syncope [12]. Prior studies have shown that ILR demonstrates greater sensitivity at detecting arrhythmias than traditional noninvasive arrhythmia detection techniques [13, 14]. In one adult study, the majority (63.9%) of tachycardia detected by loop recorder contained true tachycardia with a high sensitivity to detect induced ventricular tachycardia or ventricular fibrillation (99.3%) [15]. Another recent study showed a sensitivity of 95.4% and positive predictive value of 76.3% in the detection of atrial fibrillation with ILR [16].

Particular to our patient: the presence of two genetic variants of unknown significance and positive family history can be validated by the ILR to be significant. This can guide the geneticist to reconsider these VUS mutations to be pathologic.

*AKAP9* has been identified as a possible genetic modifier of congenital long-QT syndrome Type 1, [17]. *SCNA8A* has not been shown in the literature to be associated with sudden cardiac death or arrhythmias.

## 4. Conclusion

ILRs can play an important role in the diagnosis of life-threatening arrhythmias in children and young adults. ILR in our case showed superior diagnostic power compared to ambulatory monitors collectively with invasive EP study and exercise stress test.
